# Potential roles of mitochondrial cofactors in the adjuvant mitigation of proinflammatory acute infections, as in the case of sepsis and COVID-19 pneumonia

**DOI:** 10.1007/s00011-020-01423-0

**Published:** 2020-12-21

**Authors:** Giovanni Pagano, Carla Manfredi, Federico V. Pallardó, Alex Lyakhovich, Luca Tiano, Marco Trifuoggi

**Affiliations:** 1 Department of Chemical Sciences, Federico II Naples University, Via Cintia, 80126 Naples, Italy; 2 Department of Physiology, Faculty of Medicine and Dentistry, University of Valencia-INCLIVA, CIBERER, 46010 Valencia, Spain; 3 Vall d’Hebron Institut de Recerca, 08035 Barcelona, Spain; 4 Institute of Molecular Biology and Biophysics of the “Federal Research Center of Fundamental and Translational Medicine”, 630117 Novosibirsk, Russia; 5 Department of Life and Environmental Sciences, Polytechnical University of Marche, 60100 Ancona, Italy

**Keywords:** Sepsis, Pneumonia, *α*-Lipoic acid, Coenzyme Q10, Carnitine, COVID-19

## Abstract

**Background:**

The mitochondrial cofactors *α*-lipoic acid (ALA), coenzyme Q10 (CoQ10) and carnitine (CARN) play distinct and complementary roles in mitochondrial functioning, along with strong antioxidant actions. Also termed mitochondrial nutrients (MNs), these cofactors have demonstrated specific protective actions in a number of chronic disorders, as assessed in a well-established body of literature.

**Methods:**

Using PubMed, the authors searched for articles containing information on the utilization of MNs in inflammatory disorders as assessed from in vitro and animal studies, and in clinical trials, in terms of exerting anti-inflammatory actions.

**Results:**

The retrieved literature provided evidence relating acute pathologic conditions, such as sepsis and pneumonia, with a number of redox endpoints of biological and clinical relevance. Among these findings, both ALA and CARN were effective in counteracting inflammation-associated redox biomarkers, while CoQ10 showed decreased levels in proinflammatory conditions. MN-associated antioxidant actions were applied in a number of acute disorders, mostly using one MN. The body of literature assessing the safety and the complementary roles of MNs taken together suggests an adjuvant role of MN combinations in counteracting oxidative stress in sepsis and other acute disorders, including COVID-19-associated pneumonia.

**Conclusions:**

The present state of art in the use of individual MNs in acute disorders suggests planning adjuvant therapy trials utilizing MN combinations aimed at counteracting proinflammatory conditions, as in the case of pneumonia and the COVID-19 pandemic.

## Introduction

Acute pathological conditions display well-established links with oxidative stress (OS), through a number of different or complementary mechanistic features, as early studies have reported [1–4]. Fighting acute diseases has long been a target in medicine and over time has come to encompass a range of pharmacological and immunological tools, including the use of adjuvant means for mitigating the inflammatory conditions in relevant therapeutical strategies [5–8].

A major contemporary case of inflammatory pneumonia is presented by the global COVID-19 (SARS-CoV2) outbreak. Clinical presentations in COVID-19 include, but are not limited to, cough, fever, and acute respiratory distress sydrome, which can lead to serious complications for those with underlying cardiovascular disease, diabetes mellitus, chronic pulmonary disorders, renal disease and other co-morbidities [9, 10]. COVID-19 causes neutrophilia, lymphopenia, leukopenia, thrombopenia, and anemia as well as increased expression of systemic inflammatory proteins IL-6, C-reactive protein (CRP), innate chemokines (CXCL10, CCL2, CCL3) and the proinflammatory cytokine TNF-*α* [11–13]. A relevant involvement of mitochondrial dysfunction (MDF) in COVID-19 pathogenesis was recently reported by [14–16].

On a molecular level, the virus has several binding centers, including a transmembrane receptor for angiotensin-converting enzyme 2 (ACE-2) that facilitates viral entry into cells. The expression level of ACE-2 is increased with age [17, 18] and ACE-2 accumulates on alveolar, ciliated and goblet cells in the airways, the intestinal epithelium, cardiac cells and vascular endothelia [19, 20]. COVID-19 also exhibits genomic regions encoding the viral spike protein [21] which may attach to immunoglobulin CD147 on the surface of erythrocytes and some lymphocytes to attack the 1b-chain of hemoglobin, causing inhibition of heme metabolism [22–24]. This results in a strong OS and uncontrolled release of proinflammatory cytokines which has been termed “cytokine storm” [25].

Sepsis, on the other hand, is a pathogenesis caused by bacterial, viral, fungal, or protozoan infection, and also results in an inflammatory response and poor delivery of oxygen to tissues [26]. The most common consequences are impaired vascular permeability, cardiac malfunction, and MDF leading to impaired respiration [27]. As in COVID-19, the course of sepsis is often accompanied by a cytokine storm, leading to OS [14]. An important target of altered inflammation in the COVID-19 pathology has been shown to be also the endothelium with recent evidences indicating that the clinical condition produced by COVID-19 infection is not primarily a respiratory pathology, but rather a coagulative disorder [23, 28]. The endothelium plays a major role in the regulation of coagulative processes; thus, OS may disturb endothelial function, promoting the inactivation of beneficial endothelial-derived nitric oxide.

The relationship between OS and the risk of death in patients infected with COVID-19 suggests the need for alternative approaches to counteract this infection [28]. In addition, a recent report on the possible participation of COVID-19 in weakening mitochondrial functions suggests the need to consider these organelles as an object for adjuvant therapeutic effects targetting [16, 17, 29, 30].

In view of contributing to the mitigation of prooxidant state in COVID-19, the use of several antioxidants has been proposed, as in the case of melatonin [31], vitamin C [32], vitamin D [33], vitamin B12 and nicotinamide [34], resveratrol [35], and herbal preparations [36–38]. The rationale of these adjuvant strategies has been recenty reviewed by Quiles et al. [39].

This concurs with antioxidant therapy against sepsis that also suggests focuses on improving mitochondrial functions [40]. A range of studies has assessed the adjuvant role of three mitochondrial cofactors in mitigating a prooxidant state, with background data deriving from experimental and clinical studies.

### MDF and energy deficiency during sepsis and COVID-19

Many researchers have postulated that systemic inflammation, accompanied by elevated levels of TNF-*α*, IL-1 and PDGF, was the main determinant of the pathogenesis of sepsis and septic shock [41]. The relationship between increased production of nitric oxide, antioxidant depletion and a decrease in the activity of complex I of the respiratory chain in patients with sepsis has been well demonstrated [42, 43]. Persistent inflammation during sepsis can be caused by overproduction of mitochondrial ROS (mtROS) with consequent mitochondrial damage and MDF. Since the main role of mitochondria is to supply cells with energy, the above consequences should lead to a decrease in the synthesis of ATP. Indeed, decreased levels of ATP in the liver [44], kidney [45] and blood [46] were associated with the severity of sepsis [46, 47]. Although data on ATP levels and mitochondrial function are still emerging in COVID-19 patients, there are many reasons for drawing parallels with sepsis. In particular, the most recent work by Gibellini et al. [48] shows a decrease in ATP and MDF levels in patients infected with SARS-CoV-2. This means that mitochondria may be dysfunctional and unable to cope with the hypermetabolic demands associated with COVID-19 sepsis. Excessive ROS levels have also been seen in critically ill patients with COVID-19, indicating MDF’s involvement in the disease [49, 50]. In general, approaches targeting mtROS should be incorporated into preventive and therapeutic strategies against sepsis [7] and COVID-19-associated sepsis [51]. One such approach may be metabolic resuscitation with MNs, which can prevent uncontrolled production of mtROS and help maintain tissue homeostasis during these diseases.

### Mitochondrial nutrients: action mechanisms and antioxidant properties

Over the past several decades, a body of literature has established distinct, yet complementary, roles of MNs in mitochondrial functions [52, 53]. Comprehensive recent reviews have been focused on the roles and on the prospective potential clinical utilization application of *α*-lipoic acid (ALA) [54–56], coenzyme Q10 (CoQ10) [8, 57–60] and carnitine (CARN) [61, 62]. We have reported previously on the combined features of MDF, prooxidant state and prospective use of MNs in an extensive number of chronic, age-related or genetic disorders [6, 63–67]. Unlike chronic disorders, a relatively lesser body of literature has been focused on acute disorders, in spite of their—quite obvious—association with a prooxidant state as in, for example, sepsis.

The relative roles of each MN in counteracting acute prooxidant conditions are reported in the following tables, with data deriving from in vitro and animal studies and from clinical trials.

As shown in Table 1, in vitro studies have shown the relevance of each MN in a number of prooxidant-related conditions. Murine, rat and human cell lines, characterized by prooxidant state endpoints, were tested for antioxidant effects of ALA, which was found to inhibit signal-regulated kinase-1 (ERK1), prooxidant interleukins and other OS biomarkers [68–73]. An analogous antioxidant action was found by Schmelzer et al. [74] by testing CoQ10 in murine cells, which exerted anti-inflammatory properties via NFκB1-dependent gene expression. Further studies on models of inflammation included human endothelial cells at different levels of replicative senescence which were challenged with LPS. In this context, the reduced form of CoQ10 was particularly effective in preventing the modulation of inflammatory markers that characterize the senescence-associated inflammatory phenotype [75]. Further, CARN, when tested in rat renal cells or cardiomyocytes, was found to enhance SOD2 expression and to counteract OS and inflammation [76, 77]. Taken together, these studies of the in vitro MN-associated antioxidant effects provide a body of evidence suggesting a protective antioxidant action of MNs at the organismal level.

**Table 1:** Reports on in vitro effects of mitochondrial nutrients [MN: ALA, CoQ10 and (acyl-)CARN] focused on anti-inflammatory end points

MN	Test model	Effects	References
ALA	C_2_C_12_ myotubes	Regulating IL-6R and gp130 expression	[53]
	SK-N-BE neuroblastoma cells	Repression of IL-1b and IL-6 dependent on DNA methylation	[54]
	Murine RAW 264.7 cells	Inhibited ERK, p38 and NFκB	[55]
	Murine RAW 264.7 cells	Inhibited signal-regulated kinase-1 (ERK1) and peroxisome proliferator-activated receptor gamma (PPARγ)	[56]
	Rat embryonic fibroblasts	Decreased *β*-galactosidase, oxidative stress biomarkers, and number of apoptotic cells via the caspase-dependent pathway	[57]
	Human glioblastoma cells	Decreased apoptotic, inflammatory and oxidant effects of TRPA1 activation	[58]
CoQ10	Murine RAW 264.7 cells	Anti-inflammatory properties via NFκB1-dependent gene expression	[59]
	Human dermal fibroblasts human umbilical vein endothelial cells (HUVECs)	CoQ10-induced improvement of oxidative status via miR-146a modulation	[60]
CARN	Rat renal cells (NRK-52E)	Leptin-induced oxidative stress and inflammation were reversed by CARN	[61]
	H9c2 rat cardiomyocytes	Promotes STAT3 activation and increases the expression of SOD2	[62]

Testing of the effects of MNs in animal models of acute inflammation conditions is summarized in Table 2. A number of studies in the recent decade have tested the ALA-associated anti-inflammatory effects on rats [78–86] and mice [87, 88]. The model disorders included multiple-organ sepsis [78, 81, 82, 86], endotoxemia [79], metal or organic poisoning [81, 84], and radiation-induced damage [88]. Altogether, ALA administration was found to decrease inflammatory response, H_2_O_2_, MDA levels, myeloperoxidase activity, and cytokine levels. Thus, the body of evidence for ALA-associated anti-inflammatory actions provides strong suggestions toward the adjuvant use of this MN in counteracting inflammatory conditions.

**Table 2:** Reports on the effects of mitochondrial nutrients (MN) on anti-inflammatory endpoints tested in animal studies

MN	Species (strain)	Effects	References
ALA	Rats	Decreased kidney injury in a model of sepsis	[63]
	Wistar rats	Attenuated inflammatory response and improved multiple organ dysfunction syndrome caused by endotoxemia	[64]
	Rats	Decreased H_2_O_2_, MDA levels, and myeloperoxidase activity in ulcerative colitis	[65]
	Wistar rats	Reduced inflammation and oxidative stress in liver and kidney after sepsis	[66]
	Wistar–Kyoto rats	Counteracting counteracting gold nanoparticle-induced oxidative stress	[67]
	Sprague–Dawley rats	Decreased renal and gut injury, levels of IL-1*β*, TNF-*α*, and NO synthase	[68]
	Wistar rats	Decreased oxidative stress and the level of C-reactive protein and increased antioxidant potential in Cd-induced oxidative stress	[69]
	Ovariectomized rats	Prevented GSH and total non-enzymatic antioxidants depletion, and restored GPx and GR activities, TNF-*α*, and IL-6 in ovariectomized rats	[70]
	Rats	Decreased cytokine levels in acute respiratory distress syndrome	[71]
	Mice	Mitigated infiltration of most inflammatory cells, inflammation and vascular damage in radiation-induced pneumonitis	[72]
	C57BL/6 Mice	Decreased lipopolysaccharide-induced acute inflammatory response	[73]
CoQ10	Lewis rats	Decreased TBARS and IL-1 in methotrexate-induced rheumatoid arthritis	[74]
	Wistar rats	Protective effects on multiple organ damage and histopathologically following cecal ligation and puncture-induced sepsis	[75]
	C57BL/6J mice	Decreased NFκB phosphorylation; abrogated MDA and 8-OHDG, and restored cellular glutathione in experimental cerebral malaria	[76]
CARN	STAM mice	Prevented progression of non-alcoholic steatohepatitis by upregulating the mitochondrial *β*-oxidation and redox system	[77]
CARN	Sprague–Dawley rats	Peritonitis positively affected by CARN following puncture sepsis	[78]
	Albino Wistar rats	Proinflammatory cytokines following inflammation-induced osteoporosis	[79]
	Mice	Ameliorated liver inflammation and serum proinflammatory markers in cancer cachexia through regulating CPT I-dependent PPARγ signaling	[80]
Acetyl-CARN	Swiss Albino mice	Protective and therapeutic effect in neuroinflammation	[81]
	Wistar rats	Decreased inflammation by the overexpression of NFκB and IL-1 and IL-6 following as-induced oxidative damage	[82]

CoQ10 was also tested in rat and mouse models (Table 2), for its ability to counteract inflammatory conditions as drug-induced [89], or in puncture-induced sepsis [90], or experimental cerebral malaria [91].

Overall, CoQ10 was found to decrease MDA, TBARS and 8-OH-dG. Though through a more limited body of evidence compared to ALA, also CoQ10-associated anti-inflammatory properties may suggest the grounds for the design of adjuvant clinical treatments in acute disorders.

The animal studies of CARN- or acetyl-CARN-induced protection against proinflammatory conditions were focused on the same set of test-induced noxae (steatohepatitis, peritonitis, neuroinflammation) [92–95], as shown in Table 2. The results showed that (acetyl-)CARN decreased the levels of several proinflammatory endpoints, including proinflammatory markers, NF-ĸB and IL-1 and IL-6, and ameliorated organ inflammation [96, 97].

Thus, from the evidence provided in animal studies, each MN provides multiple means of protection against a number of proinflammatory conditions.

The reports from clinical trials on MNs in acute disorders are relatively few compared to the wealth of literature assessing the positive effects of MNs in several chronic diseases, such as type 2 diabetes and aging-related or cardiovascular disorders. An example of this growing body of literature on clinical trials in a number of chronic disorders may be found in our review [6], which cites a total of 262 reports on clinical trials testing MN-associated protective effects in patients affected by an extensive number of chronic disorders. As shown in Table 3, ALA was administered to patients admitted for hemodialysis [98], or undergoing cardiopulmonary surgery [99], or affected by ischemia—reperfusion injury [100]. Following ALA administration, patients underwent decrease in inflammatory markers, C-reactive protein (CRP), and IL-6 and IL-8 levels.

**Table 3:** Reports on the effects of mitochondrial nutrients (MN) on anti-inflammatory end points tested in clinical trials on patients with acute disorders

MN	Disease/condition	No. Of patients	Duration	Effects	References
ALA	Hemodialysis	63	8 weeks	Decreased C-reactive protein (CRP)	[83]
	Cardiopulmonary surgery	30	24 ± 9.4 months	Significantly decreased IL-6 and IL-8 levels	[84]
	Ischemia–reperfusion injury	26	> 14 days	Decreased inflammatory markers, and early kidney dysfunction and pancreatitis	[85]
CoQ10	Septic shock	14	72 h	Significantly lower CoQ10 plasma levels in septic shock patients than in healthy controls. CoQ10 negatively associated with inflammatory molecules	[86]
	Acute influenza	50	3 influenza seasons	Significantly lower CoQ10 plasma levels in patients with acute influenza infection	[87]
	Papillomavirus skin warts	156	90 days	Decreased viral load and increased antiviral cytokine levels	[88]
CARN	Hemodialysis or chronic peritoneal dialysis	113	6 months	Suppressed inflammation, CRP	[89]
	Hemodialysis	42	6 months	Decreased CRP	[90]
	Hemodialysis	36	12 weeks	Decreased CRP	[91]
	Septic shock	31	28 days	Decreased mortality	[92]
	Coronary artery disease	47	12 weeks	Decreased inflammation markers(CRP, IL-6 and TNF-*α*)	[93]
	Perioperative atrial fibrillation	134	48 h post-operation	Decreased CRP	[94]

CoQ10 levels were significantly lower in patients with acute influenza infection [101, 102]. CoQ10-supplemented patients showed decreased levels of inflammatory markers such as IL‐2 and TNF‐*α*, although no correlation with IL‐6 and IL‐10 was found [102]. Patients affected by papillomavirus skin warts and administered with CoQ10 underwent decreased viral load and increased antiviral cytokine levels [103] (Table 3).

CoQ10 was shown to improve clinical parameters as well as MDF in septic patients who received 100 mg CoQ10 twice a day for 7 days. In a randomized trial (*n* = 40), decreased levels of TNF-*α* and malondialdehyde were obtained in the early phase of septic shock patients [104].

Concurrent reports on CARN administration to patients undergoing hemodialysis [105–107], or septic shock [108] or affected by coronary artery disease [109], or perioperative atrial fibrillation [110] found CARN-induced significant decrease in CRP or decreased mortality, as shown in Table 3. The relevance of CRP in inflammation and OS had been established in early studies [111], thus the adjuvant role of CARN in mitigating a number of proinflammatory conditions should be ascertained.

### Mitochondrial nutrients: safety, and their combined administration in counteracting proinflammatory conditions

#### Safety

##### α-Lipoic acid

α-Lipoic acid is a physiological compound produced in the mitochondria as a part of their basic metabolism (Krebs cycle). Degradation of ALA is similar in humans and in rats [112], and the safety of ALA has been demonstrated in multiple clinical studies [113, 114]. Only one report of acute ALA-induced toxicity [115] was related to a suicidal attempt that was, however, reversed after a 3-d supportive treatment. Overall, a body of literature has assessed the protective action of ALA against a number of xenobiotics in in vivo and in vitro investigations [reviewed by 116].

##### Coenzyme Q10

Coenzyme Q identifies a family of lipohilic cofactors with ubiquitous presence in many organisms [117]. The most abundant form in humans is CoQ10, being characterized by a side chain consisting of ten isoprenoid units. As the other MNs considered, it is an endogenous molecule also introduced through the diet. Coenzyme Q10 is a natural—and indispensable—compound present in mitochondria (electron transport chain). The use of CoQ10 as a dietary supplement offers very low toxicity and does not induce serious adverse effects in humans [118]. CoQ10 was well tolerated at up to 900 mg/day according to Ikematsu et al. [119]. In addition, administration of exogenous CoQ10 does not inhibit the physiological production of CoQ10 [120, 121]. A recent study by Sadeghiyan Galeshkalami et al. [122] reported on the benefits of ALA and coQ10 combination on experimental diabetic neuropathy by modulating OS and apoptosis.

##### Carnitine

The amino acid derivative CARN and its active stereoisomer acetyl-CARN (ALC) have been used in a number of human studies alone or as part of a combination therapy since the early 1980s [123]. ALC is synthesized in many tissues and has low toxicity [124]. Administration of CARN in clinical studies including an extensive number of disorders (Alzheimer’s disease, depression, aging, diabetes, ischemia and other neurological diseases) did not report major toxic effects [6, 124]. Song et al. [125] performed a meta-analysis of randomized controlled trials and reported that CARN had good tolerance in patients with chronic heart failure, improving clinical symptoms and cardiac functions.

### Toward combined MN administration

**Fig. 1:**
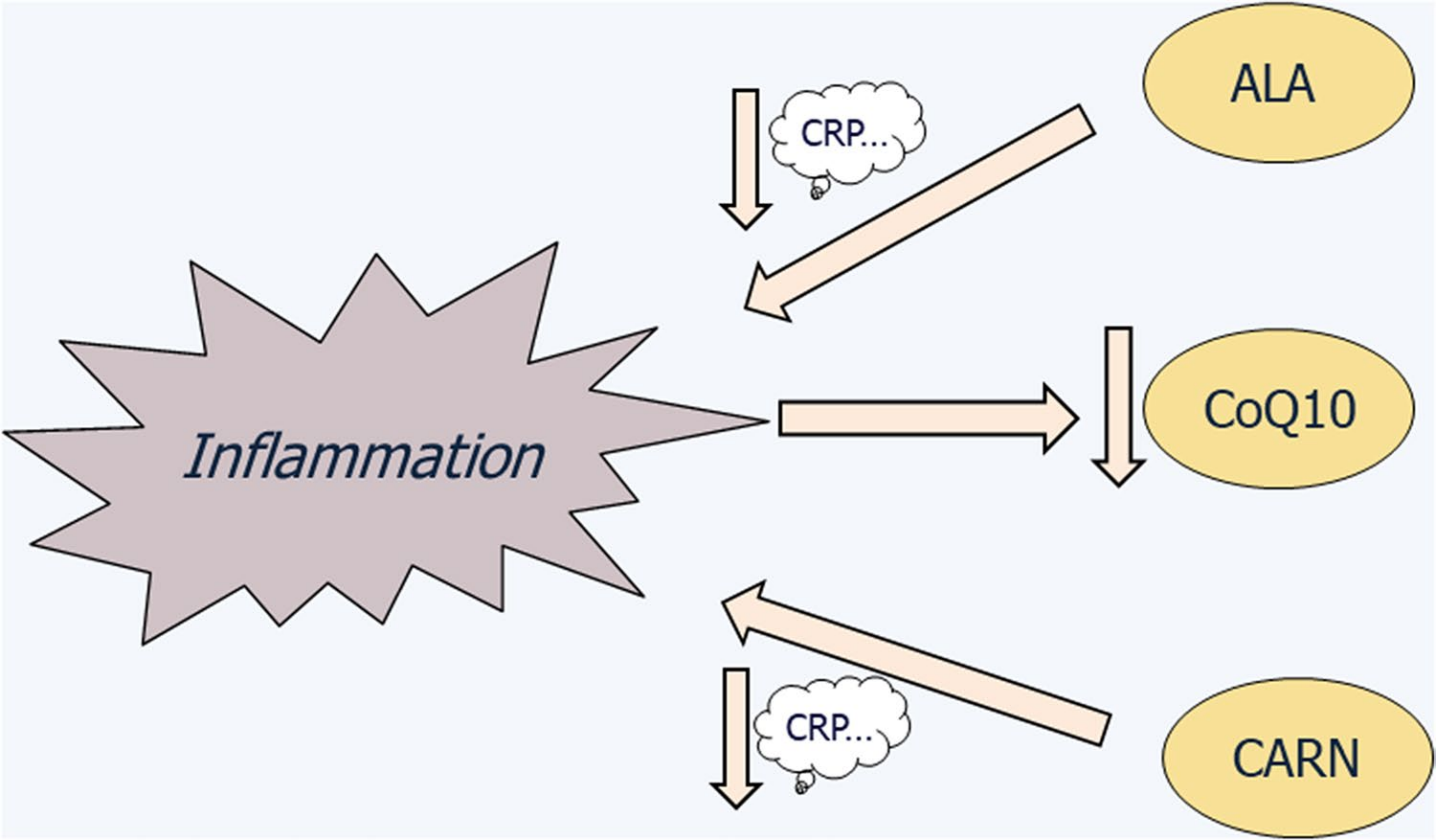
Outline of the major relationships between proinflammatory conditions and MNs, displaying decreased CoQ10 levels in plasma of patients with acute disorders, while ALA and CARN exert decreased levels of CRP and other inflammation biomarkers

Based on the evidence from experimental studies and from clinical trials, it may be concluded that separate administration of ALA, coQ10, or CARN is safe in human and in animal health. Thus, as conceptually depicted in Fig. 1, both ALA and CARN were found to lower the levels of several inflammation biomarkers, such as CRP, both in animal models [71] and in humans [100, 101, 104, 105]. Another direct link of proinflammatory conditions with MNs was provided by Donnino et al. [101] and by Chase et al. [102], who reported decreased CoQ10 plasma levels in patients affected by septic shock or by acute influenza.

A question may be raised about using individual MN administration in acute disorders, without any known attempt to test two or three combined MNs. Only a few clinical trials [122, 126, 127] investigated the effects of two combined MNs in chronic disorders, while no report is available—to the best of our knowledge—in testing three MNs concurrently.

Although there are no reports of the combined use of the three MNs in humans, any combined administration should not present potential problems when administered in patients suffering from acute disorders. According to the major and distinct interactions of MNs displayed in inflammatory conditions, as summarized in Fig. 1, it may be expected that: (a) CoQ10 administration should counteract the reported CoQ10 deficiency associated with inflammation and (b) both ALA and CARN administration should contribute to decreasing a set of inflammation biomarkers including, but not confined to, CRP. The present state-of-art is confined to clinical trials in one MN. This might be seen as a self-mutilation in the frame of adjuvant strategies targeted to mitigation of inflammatory conditions, such as sepsis, influenza, pneumonia, or other acute disorders. Taken together, the available knowledge about safety and anti-inflammatory effectiveness of each MN should prompt the combined use of these autochthonous cofactors in adjuvant therapeutic design originally designed for mitochondrial diseases [128]. The same rationale may be designed in view of mitigating acute disorders such as pneumonia infections. So far, clinical management of COVID-19 has been suggested by means of blocking cytokine storm through corticosteroids [129] or cytokine inhibitors [130, 131], controlling systemic inflammation via intravenous immunoglobulins injection [132], or inhibition of Janus kinases [133], and intervention with antimalarial drugs to inhibit tissue infection and viral replication [134]. It is worth noting that tocilizumab, as tested in COVID-19 [132, 133], is a well-established IL-6-blocking drug used in rheumatoid arthritis, both decreasing OS and MDF [135, 136].

### Working hypothesis: comparing redox potential of MNs and of other antioxidant agents

Counteracting the course of disorders characterized by a prooxidant state has been a goal of an extensive body of experimental and clinical literature (as summarized in Tables 2, 3). Apart from the attempts to utilize MNs for this purpose, a long list of natural or synthetic antioxidants, vitamins and herbal preparations has been reported in the literature focused on mitigating COVID-19 progression [31–38]. Without regarding MNs as alternative means in counteracting inflammation, one might suggest combining these agents with well-established antioxidants, such as melatonin and/or resveratrol [31, 35].

However, with regard to MNs and the broader field, a major question arises about the quantification of the redox properties of any unspecified “antioxidant”, such as redox potential. To date, attempts to accomplish this task are frustrated by the multiplicity of parameters to be considered to obtain an endpoint that may be considered valid for this purpose. These parameters, mostly obtained in physico-chemical studies, encompass a number of variables, such as temperature, pH, concentration, dimerization, and multiple free radical formation [137–139]; thus, an effort to compare the antioxidant actions of several chemicals is presently unavailable. This therefore may suggest the timeliness of a quantitative comparison of antioxidant actions, under defined—physiological—conditions such as ionic strength, pH, and temperature, which may reflect the parameters detected in basal vs*.* pathological conditions. To provide an experimental and clinical choice among the multitude of antioxidants, this investigation, as yet unaccomplished, is much warranted.

## Conclusions

The present paper reviews the experimental and clinical literature regarding the use of MNs in acute disease conditions, rather than presenting the more extensive literature about chronic disorders. The available literature provides definite evidence for the protective roles of ALA, CoQ10 and CARN in counteracting inflammation in acute disorders, such as sepsis and viral infections.

A rationale is presented for the clinical design of “triad” combinations of MNs [128] in countering the progression of acute disorders, by means of adjuvant protocols that may contribute to counteracting disease-related inflammation.

A working hypothesis is raised to achieve a comparative evaluation toward the antioxidant properties of several candidate antioxidant agents.
